# Alterations of the Intestinal Mucosal Barrier and Gut Fungal Microbiome in Asymptomatic HIV-Infected Patients

**DOI:** 10.1155/cjid/6995192

**Published:** 2024-12-09

**Authors:** Wenjie Li, Yong Qing, Qiuyue Yu, Hulian Zhang, Zhen Rang, Shuangli Li, Fan Cui

**Affiliations:** ^1^School of Medicine, University of Electronic Science and Technology of China, Chengdu, China; ^2^Institute of Dermatology, Sichuan Academy of Medical Sciences and Sichuan Provincial People's Hospital, Chengdu, China; ^3^Department of Proctology and Dermatology, Chengdu Anorectal Hospital, Chengdu, China; ^4^Department of Dermatology, Chengdu Pidu District People's Hospital, Chengdu, China; ^5^Department of Dermatology, Chengdu Xindu District People's Hospital, Chengdu, China; ^6^Department of Dermatology, The Second People's Hospital of Yibin, Yibin, China

**Keywords:** asymptomatic HIV infection, *Candida albicans*, Claudin-1, fungal secretions, gut fungal microbiota, intestinal mucosal barrier damage, mycobiome, SAP1

## Abstract

Damage to the intestinal mucosal barrier and dysbiosis of the gut microbiota are critical factors in HIV progression, reciprocally influencing each other. Besides bacteria, the fungal microbiota, a significant component of the gut, plays a pivotal role in this dysregulation. This study aims to investigate changes in the gut mucosal barrier and mycobiota during the initial stages of HIV infection, focusing on the involvement of intestinal fungi and their secretions in mucosal damage. Peripheral blood, intestinal mucosa, and fecal samples were collected from 13 asymptomatic HIV-infected individuals at the non-AIDS stage and 13 healthy controls. Assessments included colonoscopy, immune function analysis, and measurement of mucosal damage markers (LPS, I-FABP, and D-LA) and inflammatory cytokines (IL-6 and IL-18). Additionally, Claudin-1 levels in mucosal samples and fungal profiles in fecal samples were evaluated. The study found that colonic abnormalities were significantly more prevalent in the HIV group compared to healthy controls (*p* < 0.001) and Claudin-1 levels were notably lower in the HIV group (*p* <  0.001). *Candida albicans* (*p*=0.0084), its secretion SAP1 (*p*=0.023), and the levels of IL-18 (*p*=0.0016) and IL-6 (*p* < 0.001) were all significantly higher in the HIV group. CD4+ T-cell counts were positively correlated with Claudin-1 expression (*p*=0.034, *r* = 0.417). *Candida albicans* showed negative correlations with several virulence factors, while other fungi exhibited varied correlations. Additionally, Claudin-1 levels were significantly negatively correlated with *Candida albicans* (*p*=0.013, *r* = −0.668), SAP1 (*p*=0.027, *r* = −0.609), IL-18 (*p* < 0.001, *r* = −0.922), and IL-6 (*p* < 0.001, *r* = −0.920). Overall, these findings suggest that asymptomatic HIV-infected individuals have already exhibited intestinal mucosal damage in the early stage and highlight the critical role of *Candida albicans* and its secretions in early-stage intestinal mucosal barrier damage.

## 1. Introduction

The intestinal mucosal barrier restricts the invasion of pathogens and their metabolites through epithelial cells, mucins, antimicrobial proteins, and immune cells [1] and promotes the coexistence of the host and microorganisms, playing a crucial protective role for the body [2]. However, due to the high concentration of CCR5-expressing (a coreceptor for HIV) CD4+ T cells in the gut, this, in turn, makes the intestinal mucosa a primary target of HIV [3]. HIV-induced damage to the intestinal mucosal barrier leads to both local and systemic inflammatory responses in infected patients [4, 5], promoting increased intestinal permeability, which in turn exacerbates the translocation of gut microbiota, thereby continuously triggering immune activation and chronic inflammation [6], accelerating disease progression. Therefore, understanding intestinal mucosal barrier damage in asymptomatic HIV-infected individuals before progression to AIDS is critical for elucidating the early pathogenic mechanisms of HIV.

The microbial barrier is an essential component of the intestinal mucosal barrier, and changes in the gut microbiota are closely related to the stability of the intestinal mucosal barrier. Previous studies have confirmed that the composition and metabolic functions of the gut bacterial microbiota in HIV-infected individuals undergo significant alterations, affecting immune homeostasis and exacerbating chronic inflammation [7–11]. However, research on gut fungi remains limited due to the challenges in detection. Notably, changes in the gut fungal community and its products have also been associated with intestinal mucosal barrier damage, bacterial translocation, immune activation, persistent inflammation, and disease progression [12–14]. Among these fungi, *Candida albicans* is a predominant commensal fungus in the human gut, and it can transition to a pathogenic form when the healthy balance is disrupted [15]. The virulence factors of fungi, particularly secretory aspartyl proteinases (SAPs), agglutinin-like sequence 3 (ALS3), phospholipase B1 (PLB1), polystyrene adhesion enhancer protein 1 (Eap1), and hyphal wall protein 1 (Hwp1), are crucial during the colonization and invasion by *Candida albicans* [16–22]. Moreover, changes in the gut fungal community and its secretions have been associated with intestinal mucosal barrier damage in HIV-infected individuals as well, making it essential to investigate these changes.

Currently, the evaluation of intestinal mucosal damage mainly includes measuring the tight junction protein Claudin-1 in the colon to directly reflect the extent of mucosal damage [23]. Lower gastrointestinal endoscopy has also proven to be a direct and effective method for assessing the extent of mucosal damage in HIV-infected patients [24]. Indirect evaluations include determining blood levels of lipopolysaccharide (LPS) [25], D-lactate (D-LA) [26], and intestinal fatty acid–binding protein (I-FABP) [27]. Therefore, this study aims to evaluate early intestinal mucosal barrier damage and changes in the gut fungal microbiome in asymptomatic HIV-infected individuals by combining endoscopic observations with measurements from blood, intestinal mucosal tissue samples, and fecal fungal microbiome analyses. Ultimately, the goal is to explore the relationship between the fungal community and early intestinal mucosal barrier damage in asymptomatic HIV-infected patients.

## 2. Materials and Methods

### 2.1. Study Design and Population

**Figure 1:**
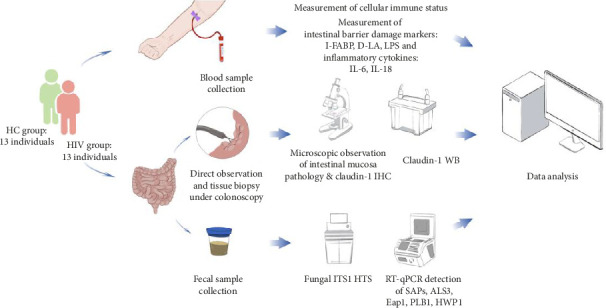
Study design. Abbreviations: HC, healthy control; HTS, high-throughput sequencing; IHC, immunohistochemistry; WB, western blotting.

Following the approval from the Ethics Committee of Sichuan Provincial People's Hospital, a prospective study was conducted from June 2023 to February 2024. First, this study selected 13 asymptomatic HIV-infected individuals who had not progressed to AIDS. These individuals were initially screened using the enzyme-linked immunosorbent assay (ELISA) and further confirmed by western blot tests at the Infectious Diseases Hospital of Chengdu, Sichuan Province. They constituted the HIV group for this research. Additionally, 13 healthy individuals were randomly chosen to create the healthy control (HC) group. The study collected data regarding age, gender distribution, modes of HIV transmission, and duration of treatment for the individuals in the treatment group (Table 1). All participants were adequately informed about the study and provided their signed consent. The peripheral blood, intestinal mucosa tissue, and fecal samples were collected from each participant (Figure 1).

Inclusion criteria for HIV-infected individuals are as follows: (1) positive for HIV antibodies and confirmed through diagnostic tests to meet the criteria for HIV infection; (2) CD4+ T cells > 200/μL, asymptomatic individuals who have not progressed to the AIDS stage, with an HIV viral load of < 40 copies/mL; (3) age between 18 and 60 years, with a normal body mass index (BMI); (4) absence of chronic infection symptoms such as fatigue, fever, night sweats, and significant weight change for at least 1 month; no metabolic diseases, chronic gastrointestinal diseases, or connective tissue diseases; no chronic diarrhea or abdominal pain symptoms in the last month; and no use of antibiotics or probiotics in the last month; (5) undergoing standard antiretroviral therapy (ART) for at least 1 year; (6) expected survival period > 2 years; (7) willing to participate in the study and signing the informed consent form; and (8) free of other sexually transmitted diseases such as syphilis and genital warts.

Exclusion criteria for HIV-infected individuals are as follows: (1) not confirmed through diagnostic tests to meet the criteria for HIV infection or HIV-infected individuals who have progressed to the AIDS stage; (2) unwilling to sign the informed consent form or do not understand the purpose of the study; (3) minors and elderly patients (age < 18 or > 60 years) and underweight or overweight individuals (BMI < 18.5 kg/m^2^ or > 23.9 kg/m^2^); (4) use of antibiotics or irregular eating habits in the last month; and (5) presence of multiorgan damage.

### 2.2. Blood Sample Collection

All enrolled participants had two tubes of fasting venous blood drawn into EDTA anticoagulant vacuum tubes, approximately 3–4 mL per tube, the morning after enrollment. One tube was used to assess the body's immune status by detecting CD4+ T-cell counts via flow cytometry. The other tube was used to indirectly assess the condition of the intestinal mucosal mechanical barrier by measuring I-FABP, D-LA, and LPS via ELISA. Additionally, serum levels of IL-6 and IL-18 were measured using ELISA to reflect the inflammatory status. After blood collection, the samples were transported to the laboratory on ice packs, with cell immune function being tested promptly (within 24 h). The blood for intestinal mucosal damage marker detection was centrifuged, and the upper layer serum was stored at −80°C for future unified testing after sample collection from all enrolled participants was completed.

### 2.3. Intestinal Mucosal Tissue Sample Collection

All enrolled participants underwent colonoscopy using the same model of colonoscope, performed by the same experienced physician. Patients were instructed to lie in a left lateral decubitus position, exposing the anus. Oxygen inhalation, electrocardiographic monitoring, and venous access were established, and after successful intravenous anesthesia, the colonoscope was inserted through the anus. Once the colonoscope reached the end of the ileum, it was advanced approximately 80 cm, then slowly withdrawn. During withdrawal, careful observation was made of the mucosa inside each colonic pouch. Biopsy specimens were taken from the distal descending colon where no localized lesions were observed, with two tissue samples collected from each study participant, each measuring approximately 0.5 × 0.5 × 0.2 cm. One of the tissue samples was fixed in 10% phosphate-buffered formaldehyde for 6 h, which would subsequently be used for paraffin embedding, sectioning, HE staining, and Claudin-1 immunohistochemical staining. The other tissue sample was immediately stored in a freezer at −80°C for future Claudin-1 protein blot experiments.

Colonic Mucosal Tissue HE and Goblet Cell Counting: (1) The fixed tissue specimens were routinely dehydrated, cleared, embedded in paraffin, and sectioned. (2) Each participant had five tissue sections randomly selected for routine histopathological analysis by the same experienced pathologist. (3) At 100x magnification, 10 fields of view were randomly selected for each section, and images were captured using the CaseViewer system. The Image-Pro Plus 6.0 software was used to count the number of goblet cells and total cell numbers within the colonic glands in each field of view, calculating the average ratio of goblet cells to the total number of cells in the colonic glands across the 10 fields of view, i.e., the goblet cell ratio.

Claudin-1 Immunohistochemical Analysis: One tissue section was randomly selected for Claudin-1 immunohistochemical analysis using the streptavidin–biotin–peroxidase complex (SP) method. The procedure was carried out in accordance with the instructions provided with the kit. The expression of Claudin-1 was analyzed using the Image-Pro Plus 6.0 image analysis software to determine its average optical density value.

Claudin-1 Protein Blotting Experiment: Claudin-1 protein was extracted from intestinal mucosal epithelial tissue, and its protein content was measured prior to performing SDS–PAGE gel electrophoresis. The process was followed by membrane transfer, blocking, incubation with primary and secondary antibodies, and then ECL chemiluminescence development. The Image J software was used to calculate the grayscale value ratio of the target protein Claudin-1 to the internal reference protein β-actin.

### 2.4. Fecal Sample Collection

The morning after enrollment, all participants used a fecal sample collector to collect two tubes of fresh feces, approximately 1 g per tube, ensuring that the collected stool did not significantly contact the external environment. One tube was designated for the analysis of the intestinal fungal microbiota and the other for the detection of intestinal fungal secretory protein gene expression. The fecal samples were stored at −80°C within 4 h of collection.

Intestinal Fecal Fungal ITS1 High-Throughput Sequencing: The cetyltrimethylammonium bromide (CTAB) method was used to extract fecal DNA from both groups, and the purity and concentration of the extracted DNA were assessed using agarose gel electrophoresis. DNA diluted to 1 ng/μL with sterile water served as the template, with the specific primers ITS5-1737F (5′-GGAAGTAAAAGTCGTAACAAGG-3′) and ITS2-2043R (5′-GCTGCGTTCTTCATCGATGC-3′) targeting the ITS1 region of fungal DNA. High-efficiency, high-fidelity enzymes were added for the polymerase chain reaction. The products recovered by the gel recovery kit were purified, followed by library preparation using a kit, and sequenced upon passing library quality control.

Fungal Microbiota Information Analysis: (1) Sequencing Data Processing: Postsequencing data were split to obtain individual sample data. Each sample's data underwent a series of procedures to yield the final analyzable effective data. (2) OTU Clustering and Species Annotation: All effective data were clustered using Uparse software, with 97% similarity as the standard, to cluster effective sequences into OTUs. Different community levels were annotated and analyzed using Qiime software against the Unite database (v7.2) (https://unite.ut.ee/). (3) Sample Complexity Analysis (alpha diversity): Alpha diversity indices PD, Chao1, Shannon, and Simpson of the fecal fungal microbiota in both groups were calculated using Qiime software. (4) Multisample Comparative Analysis (beta diversity): Bray–Curtis dissimilarities of the fecal fungal microbiota in both groups were calculated using Qiime software. (5) Analysis of Species with Significant Intergroup Differences: Intergroup differential tests and graphing were performed using R software, setting the LDA Score to 2, and LEfSe analysis was conducted using LEfSe software.

Fungal Secretory Protein Gene Detection: Fecal fungal RNA was extracted, and primers were designed in Primer5 based on sequences for the SAPs, ALS3, EaP1, PLB1, and HWP1 found in the National Center for Biotechnology Information (NCBI). This was followed by reverse transcription and finally detection via reverse transcription quantitative polymerase chain reaction (RT-qPCR).

### 2.5. Statistical Analysis

Statistical analysis was performed using SPSS software (Version 22.0) to analyze basic participant information and differences between groups. The independent samples *t*-test, Mann–Whitney *U* test, and Fisher's exact test were employed based on the distribution of data and assumptions of variance homogeneity. Correlations between indicators were evaluated using Spearman's rank correlation. For analysis of microbial community data, Qiime software (Version 1.9.1) and R software (Version 2.15.3) were utilized. Statistical significance was set at *p* < 0.05.

## 3. Results

### 3.1. Subject Characteristics

Among the basic characteristics, CD4+ T-cell counts were significantly lower in the HIV group compared to the HC group, while no other significant differences were observed (Table 1). None of the patients had experienced gastrointestinal symptoms such as diarrhea or abdominal pain in the last month, nor had they taken antibiotics, probiotics, or similar preparations during that period.

### 3.2. Colonoscopy

**Figure 2:**
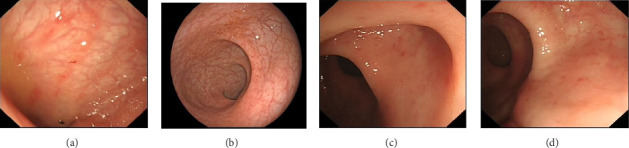
Endoscopic manifestations: (a, b) from HIV group and (c, d) from HC group.

Regarding mucosal damage, the overall prevalence of congestion, erosion, ulcers, etc., in the colorectum was significantly higher in the HIV group (69.2%) compared to the HC group (15.4%) (*p* < 0.001) (Figure 2 and Table 2).

### 3.3. Intestinal Mucosal Histopathology and Goblet Cell Counts

**Figure 3:**
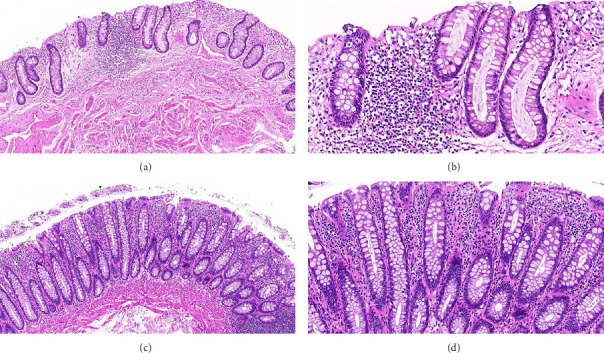
Intestinal mucosal histopathology results: (a, b) from HIV group, HE × 100, HE × 200, and (c, d) from HC group, HE × 100, HE × 200.

In the HC group, the colonic glands were regularly arranged with a high number of goblet cells, which were neatly organized. Conversely, in the HIV group, the number of colonic glands was reduced, their arrangement was less regular, and the goblet cells varied more in size and were more irregularly distributed (Figure 3). Statistical analysis revealed that the goblet cell ratio in the HIV group was significantly lower than in the HC group (*p*=0.0053) (Table 3).

### 3.4. Claudin-1 Expression

**Figure 4:**
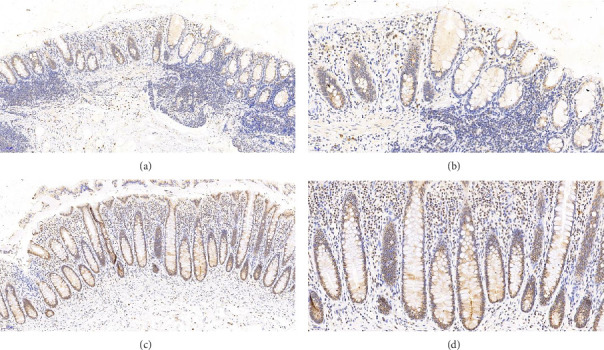
Claudin-1 immunohistochemistry results: (a, b) from HIV group, HE × 100, HE × 200, and (c, d) from HC group, HE × 100, HE × 200.

Claudin-1 Immunohistochemistry: Claudin-1 displayed notable expression in the intestinal mucosal epithelial cell membranes and cytoplasm, with positive areas appearing light yellow or brownish-yellow (Figure 4). The average optical density of Claudin-1 expression in the HIV group was significantly lower than that in the HC group (*p* < 0.001) (Table 4), as calculated using the Image-Pro Plus 6.0 software.

**Figure 5:**
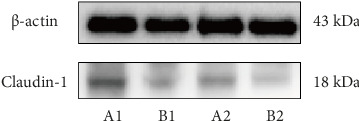
Claudin-1 protein blotting results: A1 and A2 represent the HC group, and B1 and B2 represent the HIV group.

Claudin-1 Protein Blotting: Utilizing ImageJ software to calculate the grayscale value ratio of the target protein Claudin-1 to the internal reference protein *β*-actin revealed that the relative expression level of Claudin-1 was significantly lower in the HIV group compared to the HC group (*p* < 0.001) (Figure 5 and Table 4).

### 3.5. Fecal Fungal Microbiota Sample Size, Diversity, and Composition

**Figure 6:**
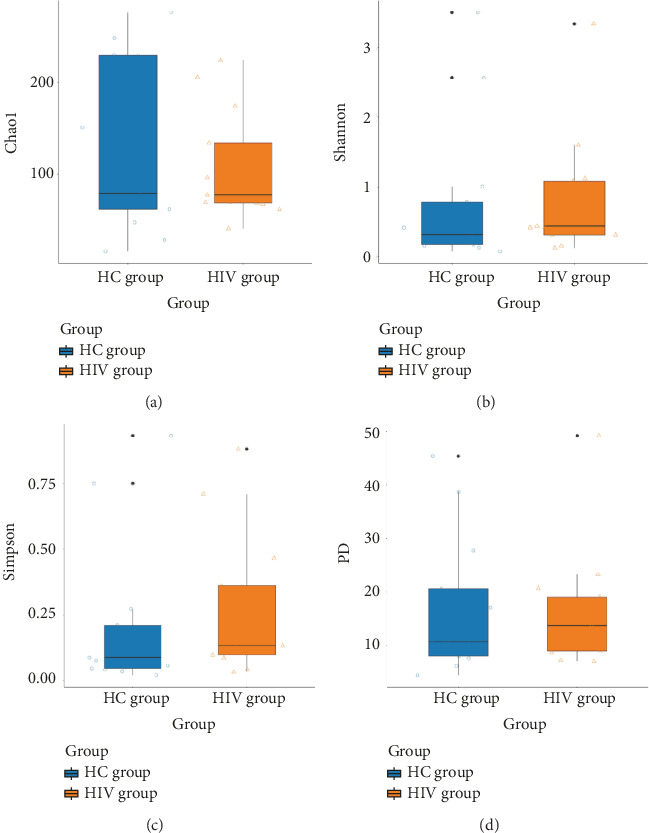
Comparison of alpha diversity between two groups: (a) Chao1 index; (b) Shannon index; (c) Simpson index; (d) PD index.

The analysis of fecal samples from both groups yielded a total of 1,482,929 effective tags with an average length of 192.88 bp. The sequencing depth was adequate to cover all fungal species present in the samples. The alpha diversity indices Chao1, Shannon, Simpson, and PD were used to characterize the species richness within the samples, revealing no statistical differences between the two groups (*p* > 0.05) (HC group vs. HIV group: Chao1: 123.6755 vs. 106.0303, *p*=0.817; Shannon: 0.7769 vs. 0.8394, *p*=0.369; Simpson: 0.2127 vs. 0.2678, *p*=0.293; PD: 16.7218 vs. 16.4063, *p*=0.644) (Figure 6).

**Figure 7:**
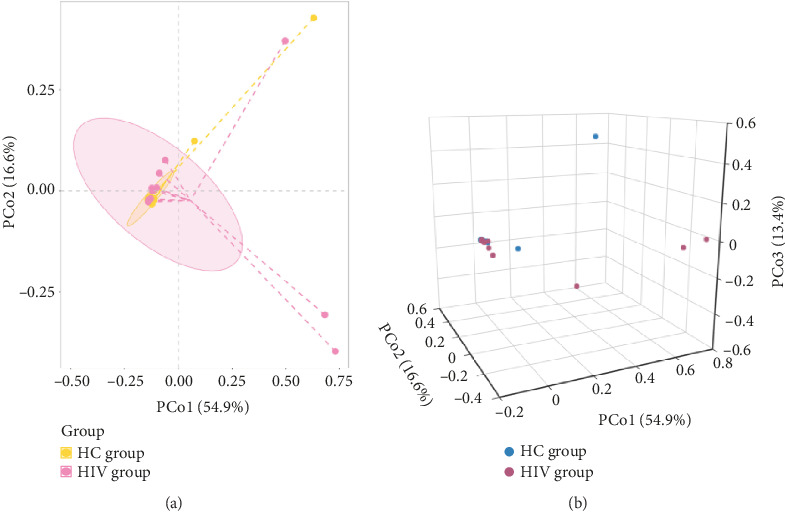
Comparison of beta diversity between two groups: (a) 2D plot and (b) 3D plot.

**Figure 8:**
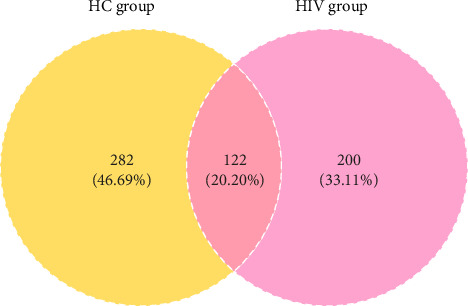
Venn diagram of unique and shared OTU counts between two groups.

Beta diversity 2D and 3D ordination plots illustrated the degree of species variation and the pattern of differences among samples. Principal coordinate analysis (PCoA) using Bray–Curtis dissimilarities showed no statistical difference between the groups (*R*^2^ = 0.0432582, *p*=0.340) (Figure 7). A Venn diagram based on the OTU abundance table highlighted the unique and shared intestinal fungal species between the groups, with the HC group having 282 unique OTUs, the HIV group 200 having unique OTUs, and 122 OTUs shared between both (Figure 8).

### 3.6. Fecal Fungal Microbiota Relative Abundance

**Figure 9:**
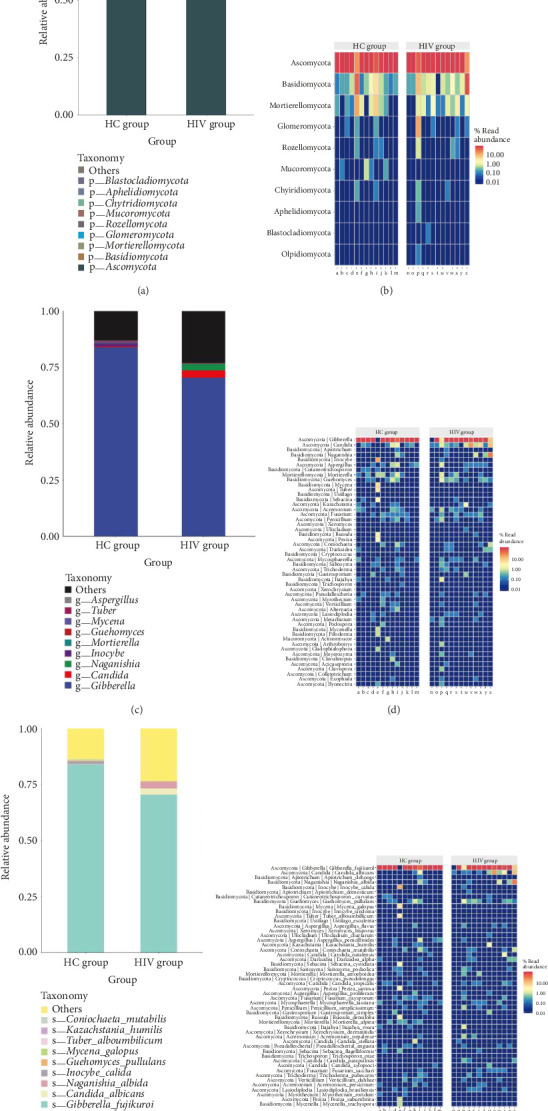
Comparison of species relative abundance bar charts and heatmaps between two groups: (a, b) phylum level, (c, d) genus level, and (e, f) species level.

At the phylum level, the intestinal fecal fungi in both groups were predominantly *Ascomycota* and *Basidiomycota*, comprising 93.63% of the HC group and 95.01% of the HIV group (Figures 9(a) and 9(b) and Table 5).

At the genus level, the HC group was primarily characterized by *Gibberella* and *Inocybe*, with a lower relative abundance of *Candida*, at 83.92%, 1.37%, and 0.29%, respectively. The HIV group was dominated by *Gibberella*, *Candida*, and *Naganishia*, with abundances of 70.41%, 3.21%, and 2.76%, respectively, indicating a significant increase in *Candida* abundance in the HIV group (*p*=0.03) (Figures 9(c) and 9(d) and Table 5).

At the species level, both groups were predominantly composed of *Gibberella fujikuroi* and *Candida albicans*, with proportions in the HC group of 83.92% and 0.14%, and in the HIV group of 70.40% and 2.84%, respectively. The abundance of *Candida albicans* significantly increased in the HIV group (*p*=0.0084) (Figures 9(e) and 9(f) and Table 5).

**Figure 10:**
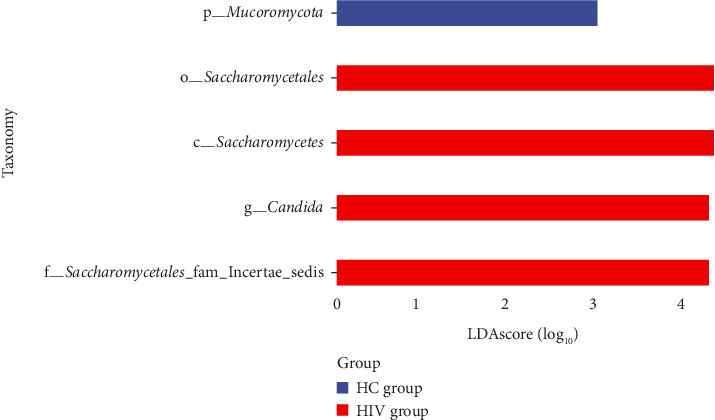
LDA value distribution histogram (LDA score set to 2).

LefSe analysis revealed significant differences in the relative abundances of the *Mucoromycota* phylum, the *Saccharomycetales* order, the *Saccharomycetes* class, and the *Candida* genus between the two groups. The *Saccharomycetales* order, the *Saccharomycetes* class, and the *Candida* genus were enriched in the HIV group, while the *Mucoromycota* phylum was enriched in the HC group (Figure 10).

### 3.7. Fungal Secretory Protein Gene Expression, Intestinal Damage Markers, and Inflammatory Cytokines

There was no expression of SAP2, SAP4, SAP6, SAP8, and SAP9 detected in samples from either group. The level of SAP1 was significantly higher in the HIV group than in the HC group (*p*=0.023), with no significant differences observed in the expression of other detected secretory proteins. There were no statistically significant differences in the levels of I-FABP and D-LA between the groups. The concentration of LPS was significantly lower in the HIV group compared to the HC group (*p*=0.016). IL-6 (*p* < 0.001) and IL-18 (*p*=0.0016) were significantly higher in the HIV group compared to the HC group (Table 5).

### 3.8. Correlation Analysis

The ratio of goblet cells to Claudin-1 expression exhibited a positive correlation in both immunohistochemistry (*p*=0.0037, *r* = 0.549) and protein blotting analyses (*p*=0.0053, *r* = 0.5303). Claudin-1 expression demonstrated a high degree of consistency between immunohistochemical and protein blotting methods (*p* < 0.001, *r* = 0.711).

Correlation analysis between Claudin-1 in the intestinal mucosa of HIV-infected patients and their cellular immune status revealed that CD4+ T-cell counts were positively correlated with Claudin-1 expression in immunohistochemistry (*p*=0.034, *r* = 0.417). However, there was no significant correlation between CD4+ T-cell counts and Claudin-1 expression in protein blotting results (*p*=0.16, *r* = 0.284).

For the correlation between inflammatory cytokines and the fungal microbiota, there was no significant correlation between *Candida albicans* species and IL-18 (*p*=0.727, *r* = 0.108) or IL-6 (*p*=0.406, *r* = 0.252). However, IL-18 was significantly negatively correlated with Claudin-1 expression in immunohistochemistry (*p* < 0.001, *r* = −0.922), and IL-6 was also significantly negatively correlated with Claudin-1 expression in immunohistochemistry (*p* < 0.001, *r* = −0.920).

**Figure 11:**
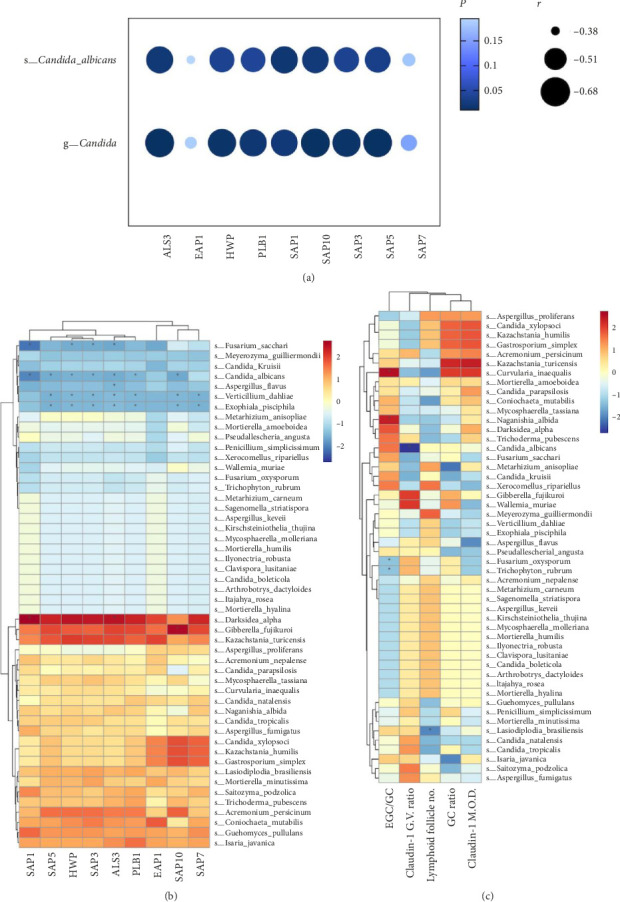
Correlation analysis of intestinal mycobiome in HIV group: (a) genus *Candida* and species *Candida albicans* with fungal metabolites, (b) species level with fungal metabolites, (c) species level with intestinal mucosal barrier function. Abbreviations: EGC, exocytotic goblet cell; GC, goblet cell; G.V., gray value; M.O.D., mean optical density.

In the HIV Group, the *Candida* genus and *Candida albicans* species demonstrated significant negative correlations with various virulence factors including ALS3, HWP, PLB1, SAP1, SAP10, SAP3, and SAP5 (*p* < 0.05) (Figure 11(a)). In addition to a notable correlation with *Candida albicans*, these virulence factors also showed significant negative correlations with *Fusarium sacchari*, *Verticillium dahliae*, and *Exophiala pisciphila* species (*p* < 0.05) and a significant positive correlation with *Darksidea alpha* species among others (*p* < 0.05) (Figure 11(b)). No significant correlations were found between peripheral blood I-FABP, D-LA, LPS, and the intestinal fungal community, nor were there significant correlations between the host's peripheral blood CD4+ T-cell counts and the intestinal fungal community.

Spearman's correlation analysis of SAP1 expression levels secreted by intestinal fungi in HIV-infected individuals with CD4+ T-cell counts did not reveal any significant correlations. However, SAP1 was significantly negatively correlated with the grayscale value of Claudin-1 (*p*=0.027, *r* = −0.609).

Correlation analysis between intestinal fungi and the condition of mucosal damage in the HIV group found: *Candida albicans* was negatively correlated with Claudin-1 G.V. ratio (*p*=0.013, *r* = −0.668); *Fusarium oxysporum* was negatively correlated with EGC/GC (*p*=0.042, *r* = −0.570); *Lasiodiplodia brasiliensis* was negatively correlated with lymphoid follicle number (*p*=0.027, *r* = −0.611); and *Naganishia albida* was positively correlated with EGC/GC (*p*=0.039, *r* = 0.577) (Figure 11(c)).

## 4. Discussion

The damage to the intestinal mucosal barrier, dysbiosis of the gut microbiome, and systemic inflammatory responses mutually exacerbate each other, creating a vicious cycle that significantly influences the pathogenesis of HIV and the occurrence of non-AIDS events [28, 29].

Previous research on the gut microbiota of HIV-infected patients primarily focused on bacterial communities. It is noteworthy, however, that fungi, though only comprising approximately 0.01% to 0.1% of the total gut microbiota, represent the second-largest component after bacteria and exhibit high variability [30, 31]. Fungi play a crucial role in regulating immune homeostasis [32] and can influence each other and the gut bacteria. In the context of dysbiosis of the intestinal mucosal barrier microbiota, the proportion of fungal colonization in HIV-infected patients is likely to increase. Furthermore, HIV-infected patients may experience persistent fungal translocation even after receiving ART, with the extent of fungal translocation depending on the level of immune suppression at the time of ART initiation [33]. Studies have identified significant alterations in the composition of the gut mycobiota in HIV-infected patients compared to healthy individuals [18, 31, 34, 35]. However, previous studies have not specifically focused on changes in gut fungi in asymptomatic HIV-infected patients nor directly linked these changes to markers of intestinal mucosal damage. This study was the first to reveal alterations in the gut mycobiota and their secretions in asymptomatic HIV-infected individuals, uncovering the potential involvement of fungi and their secretions in the process of intestinal mucosal barrier damage.

Through direct observation via colonoscopy and histopathological validation, we found that the majority of asymptomatic HIV-infected patients exhibited varying degrees of intestinal mucosal damage before progressing to AIDS. The intestinal mechanical barrier, comprising enterocytes, goblet cells, Paneth cells, and others, plays a vital role. Goblet cells, in particular, are crucial for maintaining the homeostasis of the intestinal mucosal barrier by secreting mucins and other substances that protect and repair the intestinal epithelium. Additionally, goblet cells possess antigen-presenting capabilities, making them a core component for maintaining the stability of the intestinal mucosal barrier [36]. They are also a focal point of our microscopic studies.

Furthermore, our study also revealed a significant decrease in the junctional protein Claudin-1 between intestinal mucosal cells in asymptomatic HIV-infected individuals, along with a notable increase in inflammatory factors. The junctional complexes between the cells of the intestinal mucosal mechanical barrier, including tight junctions (such as transmembrane proteins like claudins), adherens junctions (such as catenin complexes), and anchoring junctions (such as cadherins and desmogleins) [37, 38], play a crucial role in maintaining the integrity of the mucosa. Claudin-1, one of the core components of these tight junctions, has been shown to significantly influence intestinal permeability [39] and may promote apoptosis when reduced [40].

Despite the significant reduction in Claudin-1 levels in asymptomatic HIV-infected patients, we only observed a significant decrease in LPS among the indirect markers of intestinal damage, which contradicted previous studies [41–43]. We speculate that the reasons for these results might include the following: (1) the asymptomatic status of the HIV-infected individuals in our study; (2) the partial control of bacterial translocation following ART; (3) the increase in I-FABP indicating a rise in the number of intestinal epithelial cells rather than cellular damage [44]; and (4) the focal point of mucosal damage after ART being the junctional proteins rather than the intestinal epithelial cells.

In terms of changes in the alpha and beta diversity of the intestinal fungal communities, our study suggested no significant differences in the overall composition of fungi between the HIV group and the HC group. Our findings differed from previous research, and we consider the main reasons to be the following: (1) Our study population differed from previous research as we strictly included asymptomatic HIV-infected individuals, a criterion not emphasized in earlier studies, aiming to investigate changes in the gut mucosal microbiota before HIV infection progresses to AIDS and identify the early-stage triggers of mucosal damage; (2) upon comparing the baseline characteristics of the participants, we found that the average HIV infection duration, viral load levels, age, and BMI of participants in previous studies were significantly higher than those in our cohort; and (3) differences in food and nutrient intake may significantly affect gut fungal communities. The individual characteristics of asymptomatic patients, such as lifestyle and diet, may have masked changes in fungal diversity to some extent. (4) Variations in detection methods (including DNA extraction and primer selection) between our study and previous research may also account for these findings [18, 31, 35].

Moreover, our study confirmed a significant increase in *Candida albicans* in the intestines of asymptomatic HIV-infected patients compared to healthy individuals, potentially contributing to intestinal mucosal barrier damage. This observation was consistent with previous findings on alterations in the intestinal mycobiome of HIV-infected patients [31, 34, 45] and parallels observations in inflammatory bowel diseases such as Crohn's disease and ulcerative colitis, where an increase in *Candida* and *Candida albicans* has also been noted [46].

*Candida albicans*, typically a nonpathogenic commensal fungus in up to 95% of healthy individuals, can become pathogenic under disrupted physiological conditions [47, 48]. Under normal physiological conditions, commensal bacteria and *Candida albicans* compete for adhesion sites on epithelial cells, with commensal bacteria secreting short-chain fatty acids (SCFAs) and indole-3-aldehyde (IAld), while intestinal mucosal epithelial cells secrete β-defensins and LL-37. These substances play a role in inhibiting and balancing the proliferation of *Candida albicans*. During HIV infection, the overgrowth of *Candida albicans* can lead to the formation of hyphae and biofilms, disrupting the integrity of the intestinal mucosal epithelium and ultimately causing leaky gut syndrome. This leads to microbial translocation and abnormal immune activation [49].

In addition to *Candida albicans* itself, its secreted virulence factors play a crucial role in the pathogenic process. These factors, including ALS3, EAP1, HWP, PLB1, and SAPs, promote adherence, colonization, and invasion of epithelial cells, contributing to the disruption of the intestinal mucosal barrier [25, 50–55]. Particularly, the role of SAPs in intestinal mucosal damage is significant as they can degrade intestinal mucin and facilitate microbial translocation [56, 57]. Our study found significant differences in SAP1 between the two groups, suggesting that further research is needed to determine if SAP1 functions similarly to SAP2. No expression of SAP2 was detected in fecal samples of the HIV group, which might be due to the structural similarity between SAP2 and the aspartic protease of HIV-1, possibly allowing both to be inhibited by HIV-1 protease inhibitors [58]. Notably, our study found a negative correlation between the abundance of *Candida albicans* and the levels of various secreted virulence factors in HIV-infected patients, which could be attributed to enhanced adaptability and resistance of *Candida* albicans within the HIV-infected host environment [59, 60]. This relationship, along with the interactions between fungal communities and other microbial populations, merits further exploration and validation.

The transition of *Candida albicans* from a commensal to a pathogenic state is also related to a decline in host immune function [61]. However, our study did not observe this correlation. There could be several explanations for the result: (1) Our HIV cohort consisted entirely of asymptomatic individuals in the non-AIDS stage, and their condition might not have progressed to a stage with significant effects; (2) our measurements were based on CD4+ T cells in peripheral blood, whereas gut-homing CD4+ T cells are likely to be preferentially depleted during the process of HIV infection; and (3) fungal colonies constitute a small proportion of the gut microbiota, and in the process of affecting the intestinal mucosal barrier, they may play more of a participatory role rather than a dominant one.

Overall, our study demonstrates significant changes in certain fungi and their secretions in the feces of asymptomatic HIV-infected patients before progressing to the AIDS stage. These changes are closely related to intestinal mucosal damage, suggesting that intestinal fungal communities and their secretions may play an important role in the early stages of HIV infection. Altering the composition of the intestinal fungal microbiota could contribute to the treatment of HIV-infected patients. Future research should focus on the differences in intestinal fungal communities and secretions at different disease stages in HIV-infected patients, further elucidating the role of intestinal fungi in HIV disease progression. This exploration may also involve investigating the feasibility of applying techniques such as intestinal fungal transplantation in the treatment of HIV infections. However, our study has certain limitations, and future research can improve in the following areas: (1) We did not measure the degree of fungal translocation, such as determining the levels of (1 ⟶ 3)-β-D-glucan (βDG); (2) colonoscopy biopsies were limited to certain regions, which may not represent the variability across different intestinal regions; multiple biopsy sites can be considered in future studies; (3) this study did not investigate the relationship and interactions between intestinal bacteria and fungi; and (4) the patient sample size in this experiment was small, and larger-scale experiments should be considered in the future.

## 5. Conclusion

In this study, we observe that asymptomatic HIV-infected patients in the pre-AIDS stage have already suffered from damage to the intestinal mucosal barrier, along with significant alterations in some intestinal fungal flora and their secretions, accompanied by a notable increase in inflammatory factors. Notably, there is an increased abundance of *Candida albicans* and elevated levels of its secretory product, SAP1. These changes are likely involved in the process of intestinal mucosal barrier damage.

## Figures and Tables

**Table 1:** Basic characteristics of the population.

Indicators	HC group (*n* = 13)	HIV group (*n* = 13)	*p* value
Age (years)	36.62 ± 10.36	32.15 ± 9.02	0.253
Male (%)	13 (100%)	13 (100%)	—
Heterosexual transmission	—	12	—
Homosexual transmission	—	1	—
Treatment duration (months)	—	38.8 ± 10.5	—
CD4+ T-cell count (/μL)	655.77 ± 129.52	512.62 ± 178.04	0.0277

**Table 2:** Colonoscopy findings.

Colonoscopic appearance	HC group (*n* = 13)	HIV group (*n* = 13)	*p* value
Congestion only	1	4	—
Erosion	1	3	—
Ulceration	0	1	—
Polyps	0	0	—
Other lesions	0	1	—
Overall prevalence	15.4%	69.2%	< 0.001

**Table 3:** Intestinal mucosal histopathology observations.

Observation indicators	HC group (*n* = 13)	HIV group (*n* = 13)	*p* value
Number of lymphoid follicles (/HPF)	0.846 ± 0.689	2.154 ± 1.345	0.0059
Goblet cell ratio	0.594 ± 0.167	0.410 ± 0.137	0.0053

**Table 4:** Average optical density value and protein blot grayscale value of Claudin-1.

Observation indicators	HC group (*n* = 13)	HIV group (*n* = 13)	*p* value
Average optical density value	0.309 ± 0.023	0.232 ± 0.013	< 0.001
Grayscale value	0.829 ± 0.125	0.610 ± 0.141	< 0.001

**Table 5:** Fungal microbiota, fungal secretions, and indirect markers of intestinal mucosal damage.

Detection indicators	HC group (*n* = 13)	HIV group (*n* = 13)	*p* value
*Fungal microbiota*			
p__*Ascomycota*	89.22%	87.75%	0.44
p__*Basidiomycota*	4.41%	7.26%	0.20
g__*Gibberella*	83.92%	70.41%	0.21
g__*Inocybe*	1.37%	0.01%	0.65
g__*Candida*	0.29%	3.21%	0.03
s__*Gibberella fujikuroi*	83.92%	70.40%	0.20
s__*Candida albicans*	0.14%	2.84%	0.0084

*Fungal secretions*			
ALS3	7.399 ± 12.457	7.140 ± 13.163	0.9557
EAP1	8.876 ± 17.967	6.238 ± 16.303	0.1853
HWP	7.131 ± 10.977	7.542 ± 15.079	0.3933
PLB1	7.162 ± 11.907	6.716 ± 12.266	0.7044
SAP1	5.851 ± 8.319	13.665 ± 17.794	0.023
SAP10	7.656 ± 13.458	6.737 ± 9.920	0.8804
SAP3	7.026 ± 10.751	4.921 ± 8.661	0.190
SAP5	7.099 ± 10.780	4.059 ± 7.519	0.0695
SAP7	7.947 ± 13.912	15.775 ± 51.898	0.365

*Indirect markers of intestinal mucosal damage*			
I-FABP (pg/mL)	1060.93 ± 235.18	936.47 ± 237.62	0.192
D-LA (pg/mL)	1502.47 ± 374.57	1248.53 ± 275.80	0.061
LPS (pg/mL)	1240.08 ± 476.67	859.06 ± 230.93	0.016

*Inflammatory cytokines*			
IL-6 (pg/mL)	1.37 ± 0.91	3.46 ± 1.28	< 0.001
IL-18 (pg/mL)	258.89 ± 110.03	472.19 ± 180.21	0.0016

## Data Availability

The datasets generated during the current study are not publicly available due to confidentiality of participants' information.

## Nomenclature

ART: Antiretroviral therapy
LPS: Lipopolysaccharide
D-LA: D-lactate
I-FABP: Intestinal fatty acid–binding protein
SAPs: Secretory aspartyl proteinases
ALS3: Agglutinin-like sequence 3
PLB1: Phospholipase B1
Eap1: Polystyrene adhesion enhancer protein 1
Hwp1: Hyphal wall protein 1
IL: Interleukin
HC: Healthy control
BMI: Body mass index
GALT: Gut-associated lymphoid tissue (GALT)
SP: Streptavidin–biotin–peroxidase complex
CTAB: Cetyltrimethylammonium bromide
NCBI: National Center for Biotechnology Information
RT-qPCR: Reverse transcription quantitative polymerase chain reaction
PCoA: Principal coordinate analysis
EGC: Exocytotic goblet cell
GC: Goblet cell
G.V.: Gray value
M.O.D.: Mean optical density
